# Pore-Rich Cellulose-Derived Carbon Fiber@Graphene Core-Shell Composites for Electromagnetic Interference Shielding

**DOI:** 10.3390/nano13010174

**Published:** 2022-12-30

**Authors:** Yadong Yang, Caichao Wan, Qiongtao Huang, Jun Hua

**Affiliations:** 1 College of Science, Xi’an University of Architecture and Technology, Xi’an 710055, China; 2 College of Materials Science and Engineering, Central South University of Forestry and Technology, Changsha 410004, China; 3 Yihua Lifestyle Technology Co., Ltd., Huaidong Industrial Zone, Lianxia Town, Chenghai District, Shantou 515834, China

**Keywords:** cellulose, carbon fiber, graphene, core-shell materials, electromagnetic interference shielding

## Abstract

Because of serious electromagnetic pollution caused by the widespread use of radio frequency equipment, the study of electromagnetic interference (EMI) shielding materials has been a long-standing topic. Carbon fiber and graphene composites have great potential as EMI shielding materials due to their unique microstructure and electrical conductivity. In this work, a novel kind of core-shell composite is fabricated based on the pore-rich pine needles-derived carbon fibers (coded as PNCFs) core and the graphene shell. The pore-rich PNCFs are created by KOH activation, and the integration between the pore-rich PNCFs and the graphene relies on a plasma-enhanced chemical vapor deposition (PECVD) method. The conductivity of the pore-rich PNCFs@graphene core-shell composite reaches 4.97 S cm^−1^, and the composite has an excellent EMI shielding effectiveness (*SE* > 70 dB over X-band (8.2–12.4 GHz)) and achieves a maximum value of ~77 dB at 10.4 GHz, which is higher than many biobased EMI shielding materials in the recent literature. By calculation and comparison, the large absorption loss (accounting for 90.8% of total loss) contributes to reducing secondary radiation, which is quite beneficial for stealth uses. Thus, this work demonstrates a promising design method for the preparation of green high-performance composites for EMI shielding and stealth applications (such as warcrafts, missiles, and stealth wears).

## 1. Introduction

With the rapid development of electronic information technology, radio frequency equipment has been widely used in various fields such as communications, biological medicine, and remote sensing. Radio frequency equipment brings us convenience, but it also brings serious electromagnetic pollution. Electromagnetic pollution will not only disturb the normal operation of precision instruments, but also cause harm to people’s health. Therefore, there is an urgent need to develop high-performance electromagnetic interference (EMI) shielding materials [1,2,3,4].

EMI shielding materials can be roughly divided into three categories: carbon materials, metals, and conductive polymers. Among them, carbon materials have suitable electrical conductivity, strong oxidation resistance, and superior thermal stability. They are quite suitable for electromagnetic shielding materials. As people’s awareness of environmental protection continues to increase, more and more attention has been paid to green chemistry and materials [5,6]. Graphite and petroleum coke-derived carbon materials are non-renewable and contaminated. Biomass, including renewable crops or animals, can be considered an environmentally friendly precursor that can be sustainably produced on a large scale and at low prices. Moreover, due to its structural diversity and tunable physical/chemical properties, biomass can be formed into specific porous and layered carbon materials by simple methods [7,8]. In recent years, biomass-derived carbon materials have been widely used for electrochemical energy storage [9,10], catalytic adsorption [11], and biosensing [12], but the development of biomass-based derived carbon for electromagnetic shielding materials is still in the early stage. For example, Shen et al. [13] prepared aerogel (Cs)/epoxy EMI shielding composites by carbonizing natural wood to obtain Cs and then backfilling them with epoxy resin to achieve an electromagnetic shielding effectiveness of 28 dB at X-band. Li et al. [14] prepared the aerogel-like carbon (ALC) based on sugarcane, and the average EMI shielding effectiveness of ALC in the X-band is 51 dB. Zhang et al. [15] employed the cotton fabric as the skeleton, and the polyethyleneimine/phytic acid (PEI/PA) layer and silver nanowires (AgNWs) conductive networks are formed on the surface of cotton fibers through layer-by-layer assembled technology and the dip-coating method. The strong electrostatic interaction and hydrogen bonding effect play key roles in their interface bonding. This kind of multilayer-coated cotton fabric is flame-retardant and has an EMI shielding effectiveness of 32.98 dB in the X-band. Pine trees are widely distributed throughout the world due to their environmental adaptability, while pine needles exhibit a rich stratification of pores. In most cases, the dropped pine needles are discarded and decay into the soil. There is evidence that pine needle-derived porous carbon has excellent electromagnetic wave absorption properties [16]. Further studies on the electromagnetic shielding properties of pine needle-derived carbon are necessary.

Meanwhile, carbon nanomaterials (carbon fibers, carbon nanotubes, graphene, etc.) have attracted the interest of outstanding scholars in different fields in the past few years due to their superior various performance. For example, Zhang et al. [17] prepared self-supporting x Ni-WC@CFP catalysts with fold-like structures using carbon fiber paper (CFP) as the carbon source by a one-step molten salt method. This Ni-doped WC-based self-supporting electrocatalyst exhibited excellent catalytic performance for efficient hydrogen production on the CFP surface by taking advantage of properties such as high specific area and high conductivity of CFP. Luo et al. [18] recently reported a new type of stretchable polymer carbon nanotube (CNF) composite electrode. Due to the excellent electrical and mechanical properties of CNF, the composite electrodes embedded with long CNF not only form an excellent conductive network (low sheet resistance of 30∼50 Ω/sq) but also have suitable tensile properties (recoverable stretching rate up to 200%) and excellent stability (over 20,000 bending and stretching cycles). The excellent work of A. Mohammadnejad et al. [19] also showed that CNT has a significant positive effect on Ni structure and mechanical properties. The authors demonstrate that the introduction of CNT can cause lattice deformation of the alloy and simplify the diffusion of alloying elements, which can accelerate the sintering kinetics. It was also demonstrated that the addition of CNT could improve the hardness and ductility of the alloy. Graphene, one of the most basic elements of carbon materials, has a perfect hybrid structure and a large conjugated system whose π-electron network can offer suitable conductive channels and reaches fast electron mobility at room temperature of 15,000 cm^2^ V^−1^ S^−1^ [20,21,22,23]. As a result, graphene has aroused great interest in the field of EMI shielding. It is believed that combining graphene with other EMI shielding active materials can realize a striking EMI shielding effectiveness because of the synergistic effects between them. In addition, developing a smart 3D structure has been demonstrated as a portal factor for the improvement of EMI shielding effectiveness due to the reinforced multiple reflection effects in the complex micro-nano structure. Xu et al. [24]. Silkworm cocoon–co-graphene (SC-Co-G) composites were prepared using cocoons as precursors of biomass carbon, which exhibited a maximum electrical conductivity of about 0.22 S cm^−1^ and electromagnetic shielding effectiveness is 55.0 dB at 12.4–18 GHz. The porous structure will greatly increase the specific surface area of the materials, and the specific surface area of the shielding materials is also a key parameter that affects the EMI shielding performance. For instance, Chen et al. [25] prepared a graphene@polydimethylsiloxane composite three-dimensional porous material using a three-dimensional nickel foam template. Graphene formed a three-dimensional conductive path. When the thickness of the composite is 2 mm or 3 mm, their EMI shielding effectiveness is 26~27 dB or 36~37 dB, respectively. Moreover, in our recent work [26], we have successfully constructed a sandwich nano heterostructure composed of carbon fibers, metallic nickel nanoparticles, and dandelion-like graphene. This top-down structural design method enhances the electromagnetic shielding effect of the material with the help of the micro-nano structural characteristics and synergy of these three components, and the final X-band electromagnetic shielding effectiveness reaches 50.6 dB. This work also proved that graphene nanosheets and carbon fibers can construct a dense conductive network and supply many mobile charge carriers and conductive channels. The electromagnetic shielding effectiveness of the 2-mm-thick composite in the X-band is as high as 58.4 dB. However, it is still meaningful to further construct a new smart nanostructure of graphene-based hybrid materials for the development of high-performance EMI shielding products.

**Figure 1:**
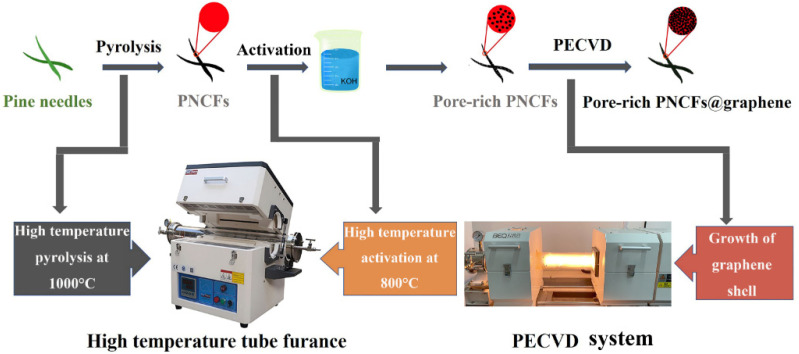
Schematic illustration of the design philosophy and synthetic strategy of the pore-rich PNCFs@graphene.

Herein, we use pine needles as raw materials and carbonize them into carbon fibers (coded as PNCFs) to integrate with graphene after an activation treatment. The activation treatment is conducted by a combined process of KOH immersion and calcination. The growth of graphene is carried out on the surface of pore-rich PNCFs by virtue of plasma-enhanced chemical vapor deposition (PECVD) [27,28]. Figure 1 depicts the preparation process of the pore-rich PNCFs@graphene core-shell composite. This core-shell composite can achieve a maximum EMI shielding effectiveness of ~77 dB in the X-band, higher than its precursor (pore-rich PNCFs, 52 dB). Moreover, the absorption shielding effectiveness (*SE*_A_) and reflection shielding effectiveness (*SE*_R_) of the pore-rich PNCFs@graphene account for 90.8% and 9.2% of the total effectiveness, respectively, revealing an absorption-dominate EMI shielding mechanism. In addition, a possible mechanism analysis on the EMI shielding process is proposed.

## 2. Experiment

### 2.1. Materials

KOH (85% purity), ethanol (98% purity), distilled water, hydrochloric acid, pure paraffin, high-purity hydrogen (99.999% purity), argon (99.9% purity), and methane (99.999% purity) are provided by Hunan FaLanKeEr Chemical Co., Ltd., Changsha, China and directly used without further purification.

### 2.2. Pyrolysis of Pine Needles

Green and fresh pine needle raw materials were washed with distilled water and then placed in an oven at 60 °C for 12 h. Then, the dried pine needles were placed in a tube furnace under N_2_ atmosphere and heated to 500 °C at a heating rate of 5 °C min^−1^, and heated at this temperature for 1 h. Subsequently, the material was heated to 1000 °C min^−1^ at a heating rate of 5 °C min^−1^, and maintained at this temperature for 2 h. Finally, the temperature was reduced to 500 °C min^−1^ at a cooling rate of 5 °C min^−1^, and finally cooled to room temperature naturally.

### 2.3. Mechano-Chemical Activation of PNCFs

A combined method of KOH immersion and calcination was used to make pores for the pyrolyzed pine needles. First, 50 mL of KOH solution was used to soak the PNCFs for 24 h (the mass ratio of PNCFs to KOH is 1:4). After being dried at 105 °C to remove moisture, the PNCFs and powder-like KOH were transferred to a nickel ark for heat treatment. The temperature was first raised from room temperature to 800 °C at a heating rate of 10 °C min^−1^ under the protection of argon gas (flow rate 250 scc/min) and then kept at 800 °C for 5 h. After that, the sample was naturally cooled to room temperature. Then the sample was washed with 10% HCl until the pH value approached 7. Then the sample was dried at 60 °C.

The activation mechanism using KOH as the activator can be described as follow. During the heating process, the KOH crystallizes into small particles and intercalates into PNCFs. When the temperature rises to 800 °C, the activator KOH reacts with PNCFs, leading to the production of K_2_CO_3_, K_2_O, etc. Subsequently, these products further react with PNCFs. These reactions not only produce abundant new micropores but also enlarge the specific surface area [29]. The relevant reactions are presented as follows:

(1) $$6KOH+2C↔2K+3H_{2}+2K_{2}CO_{3}$$

(2) $$K_{2}CO_{3}+C↔K_{2}O+2CO$$

(3) $$K_{2}O+C↔2K+CO$$

The chemically activated samples were placed in a 250 mL WC lined vial of the planetary carrier and ground using a 10 mm diameter WC ball with a ball-to-powder mass ratio set at 4:1. Milling was performed at 300 rpm for 1.5 h. Then sieve the obtained powder to remove powder agglomerates larger than 325 microns in diameter (sieve size = 0.045 mesh) in order to obtain particles of micron size. Most of the resulting pore-rich PNCFs particle sizes are distributed between 50 and 325 µm.

### 2.4. Growth of Graphene by PECVD

The growth of graphene by PECVD follows our previously reported method [28]. For the PECVD process, the CH_4_/Ar flow rate ratio and the total gas pressure were set as 20 sccm/80 sccm and 400 Pa, respectively. The substrate bias voltage was 30 V and the RF power was 200 W. The graphene was deposited at 800 °C, and the deposition time was 60 min.

### 2.5. Preparation of Paraffin/Pore-Rich PNCFs@Graphene Composites

The paraffin was first melted at 80 °C for 20 min. The liquid-stated paraffin was mixed with the pore-rich PNCFs@graphene at a weight ratio of 1:1. After the homogeneous mixing under a gentle magnetic stirring at 80 °C, the paraffin/pore-rich PNCFs@graphene composites were transferred to a mold for compression molding and naturally cooled to room temperature. The thickness of the sample was controlled at 2.000 ± 0.020 mm.

### 2.6. Characterization

Micromorphology observations were made on a scanning electron microscope (SEM, Hitachi S4800, Hitachi Limited, Tokyo, Japan). Before the observations, the samples were adhered to the sample stage and sprayed with gold for 45 s by using an Oxford Quorum SC7620 sputter coater (Quorum Co., Oxford, UK)at 10 mA (the thickness of the sputtered gold coatings is ~5 nm). SEM digitized photographs were taken at an acceleration voltage of 15 KeV with a magnification range between 75 and 10,000 g. The chemical composition of carbon nanomaterials grown by PECVD was analyzed by energy dispersive spectroscopy (EDS). The crystal structure was studied by X-ray diffraction (XRD, Bruker D8 Advance TXS, Billerica, MA, USA) with Cu Kα (target) radiation (λ = 1.5418 Å). The scan rate and scan range were set as 4° min^−1^ and 5–90°, respectively. Raman analysis was performed on a Raman spectrometer (Renishaw inVia, London, UK) employing a helium/neon laser (633 nm) as the excitation source. The instrument for tensile tests was an electronic universal material testing machine (Model CMT6104, Metz Industrial Systems., Eden, MN, USA). Transmission electron microscope (TEM) images were acquired using an FEI, Tecnai G2 F20 TEM (FEI Co., Hillsboro, OR, USA) with a field-mission gun operating at 200 kV. Atomic force microscopy (AFM) images were performed by Bruker Dimension Icon scanning probe microscope (Bruker Co., Billerica, MA, USA). The samples were ground by ball mill (PML 10). The size distribution of synthesized particles (100 mg) using a laser particle sizer (Malvern Mastersizer 2000, Malvern City, UK).

### 2.7. DFT for Analyzing the Pore Size Distribution

Seaton et al. [30] first proposed the density functional theory (DFT) in 1989, coupled with Monte Carlo (MC) molecular simulations, to calculate pore size distribution more precisely from adsorption isotherms. The DFT allows for the description of the adsorption and phase behavior of fluids in pores at the molecular level and obtains an accurate pore size distribution over the complete micro- and mesopore range. DFT provides a microscopic model of adsorption, which reflects pore size distribution more accurately than traditional thermodynamic methods (including BJH, HK, and SF).

#### 2.7.1. Adsorption Experiment

The pore structure and surface area were analyzed by N_2_ adsorption–desorption tests using an accelerated surface area and porosimeter system (3H-2000PS2 unit, Beishide Instrument S&T Co., Ltd., Beijing, China) at 77.4 K. High-purity nitrogen (99.99%) was used. Sample density was controlled at 1.000 g/cm^3^. All samples were degassed at 300 °C for 2 h prior to measurements.

#### 2.7.2. DFT Calculation Method

The International Organization for Standardization (ISO) recommends the use of DFT to calculate the pore size distribution [31]. The theory relies on the pore size distribution fundamental relationship with the experimental adsorption isotherm, *V*, given in the form of the following adsorption integral equation [32]:

(4) $$V\left(p\right)=\int_{\alpha }^{\beta }\rho \left(p,w\right)f\left(w\right)dw$$

where *V*(*p*) denotes the volume adsorbed as a function of pressure, and *ρ*(*p*, *w*) is the model adsorption isotherm given in terms of the adsorbate density calculated for model pores as a function of pore width (*w*) and equilibrium pressure (*p*). The pore size distribution, *f*(*w*), is the distribution of pore volumes as a function of pore width. The lower and upper limit of the integration range are the widths of the smallest and largest pores (where the pore width is the distance between the nuclei of C atoms on opposing pore walls) [33]. In this approach, the individual pore model isotherm *ρ*(*p*,*w*) comes from the molecular model for nitrogen adsorption on the basis of nonlocal or smoothed density approximation, and it is also the kernel file in the DFT software we use.

In this study, the DFT pore distribution model was calculated by using the ASIQwin software released by Quantachrome Instruments (Boynton Beach, FL, USA), and the model (kernel) “N_2_@77K on carbon materials (slit-shaped/spherical pores, NLDFT method, calculated using desorption curves)” was selected.

### 2.8. EMI Shielding Effectiveness Tests

A PNA-X network analyzer (N5244a, Palo Alto, CA, USA) was used for EMI shielding tests in the frequency range of 8.2~12.4 GHz (X-band) by coaxial method. The measuring device consists of two coaxial adapters (sample holders) and a network analyzer. The signal was coupled and transmitted through the coaxial cable and the attenuator. The paraffin/pore-rich PNCFs@graphene mixture was pressed into a ring mold (outer diameter: 7.0 mm, inner diameter: 3.0 mm, and thickness: 2.0 mm) and tightly fixed in the coaxial sample holder. Based on the coaxial transmission theory, the electromagnetic wave was subjected to multiple reflections and transmissions in EMI shielding samples, hence leading to the electromagnetic energy attenuation [34]. After multiple reflection and transmission of electromagnetic wave, the reflection parameters *S*_11_ and transmission parameters *S*_21_ were measured. From the *S*-parameters, we can calculate the reflection coefficient (*R*), transmission coefficient (*T*), and absorption coefficient (*A*). The reflection loss (*SE*_R_), absorption loss (*SE*_A_), and total shielding efficiency (*SE*_T_) can be calculated by Formulas (5)–(10).

(5) $$R={\left|S_{11}\right|}^{2}$$

(6) $$T={\left|S_{21}\right|}^{2}$$

(7) A=1−R−T

(8) $$SE_{A}=-10\log_{10}\left(1-R\right)$$

(9) $$SE_{R}=-10\log_{10}\left(T/1-R\right)$$

(10) $$SE_{T}=-10\log_{10}\left(T\right)$$

## 3. Results and Discussion

### 3.1. Morphology Observations and Elemental Analysis

**Figure 2:**
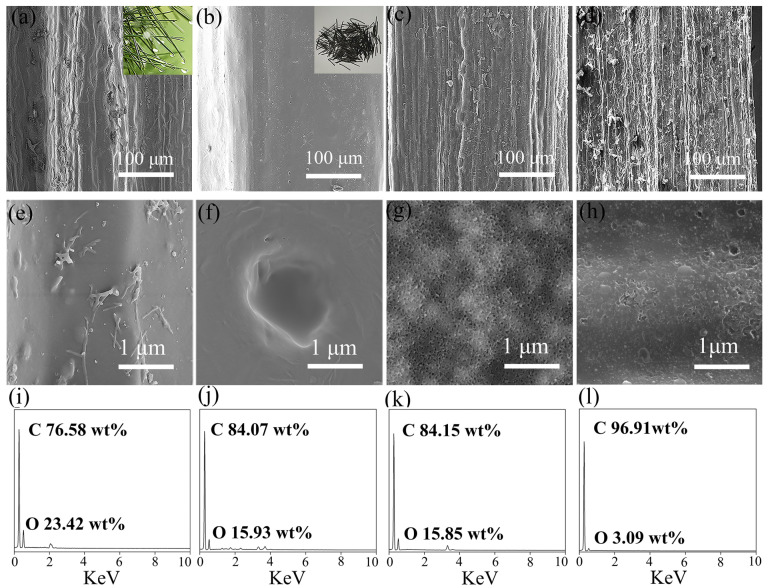
(**a**,**e**,**i**) SEM images and EDS spectrum of the original pine needles; (**b**,**f**,**j**) SEM images and EDS spectrum of PNCFs; (**c**,**g**,**k**) SEM images and EDS spectrum of the pore-rich PNCFs; and (**d**,**h**,**l**) SEM images and EDS spectrum of the pore-rich PNCFs@graphene.

The microstructure of the pore-rich PNCFs@graphene composite and its precursors is observed by SEM. As shown in Figure 2a,b, the pyrolysis treatment transforms the rough surface of pine needles to the smooth surface of PNCFs. The surface color of pine needles changes from green to black, revealing the formation of carbon components. After the activation treatment, the surface of PNCFs becomes rough again (Figure 2c), and the surface of pore-rich PNCFs becomes rougher after the growth of graphene by PECVD (Figure 2d). As can be seen from Figure 2e–g, under higher magnification of SEM images, there are no micropores on the surface of the original pine needles and PNCFs. The surface of PNCFs that has undergone pore formation treatment is covered with uniformly distributed micropores. After depositing graphene using PECVD on the pore-rich PNCFs substrate for 60 min, the surface of the pore-rich PNCFs is covered with a film containing graphene nanosheets, as shown in Figure 2h. Therefore, a binary core-shell structure pore-rich PNCFs@graphene is created. Studies have shown that the junction interface and defects between two different nano-carbons can cause charge delocalization that will favor the attenuation of electromagnetic energy [35,36]. In addition, for heterogeneous and concentric nanomaterials, their activity is higher than the activity of their individual components [37,38]. Cleverly adjusting the core and shell of these materials can produce core-shell nanostructures with customizable properties, which play a key role in EMI shielding. Graphene’s perfect two-dimensional structure, high conductivity, and impermeability to oxygen and water molecules will be the ideal “shell” choice. In addition, graphene as a shell can enhance the electrical conductivity and electrical polarization of the material, which will help improve the electromagnetic shielding ability of the composite. The chemical composition of the original pine needles PNCFs, pore-rich PNCFs, and pore-rich PNCFs@graphene is analyzed by EDS. Figure 2i–l show that all the C element signals are very strong. In addition, the signal of the O element can also be detected. The C mass fraction on the original pine needles, PNCFs, pore-rich PNCFs, and pore-rich PNCFs@graphene surface reaches up to 76.58%, 84.07%, 84.15%, and 96.91%, respectively. The increase in C mass fraction from 76.58% to 84.07% proves that the pyrolysis converts the oxygen-containing component of the pine needles into the carbon component of PNCFs, and the subsequent increase in the C mass fraction from 84.15% to 96.91% is attributed to the introduction of graphene by using PECVD.

**Figure 3:**
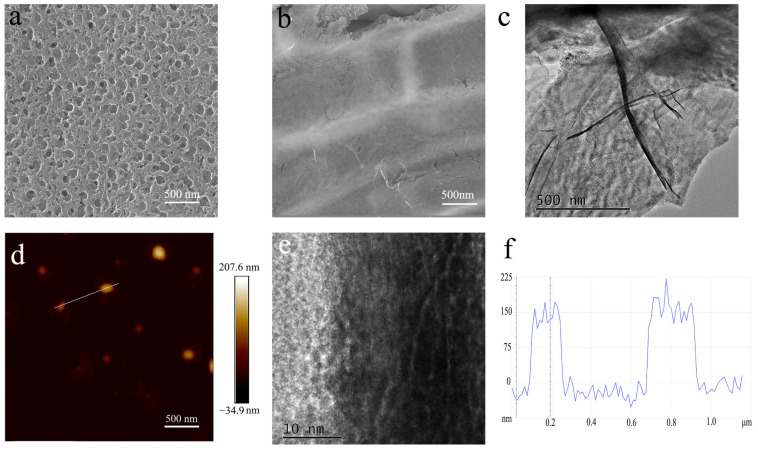
(**a**,**b**) SEM images of pore-rich PNCFs before and after PECVD; (**c**,**d**)TEM images of the graphene shell in the pore-rich PNCFs@graphene; (**e**) AFM image of the graphene shell; (**f**) cross-section analysis is based on the chosen line.

The graphene shell plays a key role in EMI shielding. As a result, we study its microstructure through SEM and AFM observations. Figure 3a,b show representative SEM images of pore-rich PNCFs before and after PECVD processing. After the growth of graphene by PECVD, the apparent graphene wall on the pore-rich PNCFs surface demonstrated the generation of core-shell structures. As shown in Figure 3c, the TEM observation shows large (micron- and submicron scale) sheets at the pore-rich PNCFs@graphene edge with wrinkled and scrolled structure, which is the typical feature of graphene. From the higher-magnification TEM image (Figure 3d), there are distinct multilayers at the edge of graphene sheets. The thickness, measured from the height probe of the AFM images (Figure 3e,f), is about 150 nm. Toward materials with low loss factor, the reflection and absorption are both limited, which need larger thickness for high EMI shielding effectiveness (*SE*). It also involves the skin depth *δ* where electromagnetic wave energy decreases to 1/e of the incident wave, described as *δ* = (π*fμσ*)^−1/2^ (*μ* = *μ*_0_*μ*_r_, *μ*_0_ = 4π × 10^−7^ H/m). In X-band (8.2–12.4 GHz), the calculated *δ* is in the range of 45.0–55.4 nm, which is much lower than the thickness of the graphene shell. Therefore, the graphene shell can effectively shield electromagnetic waves.

### 3.2. Crystal Structure, Chemical Composition, and Pore Structure

**Figure 4:**
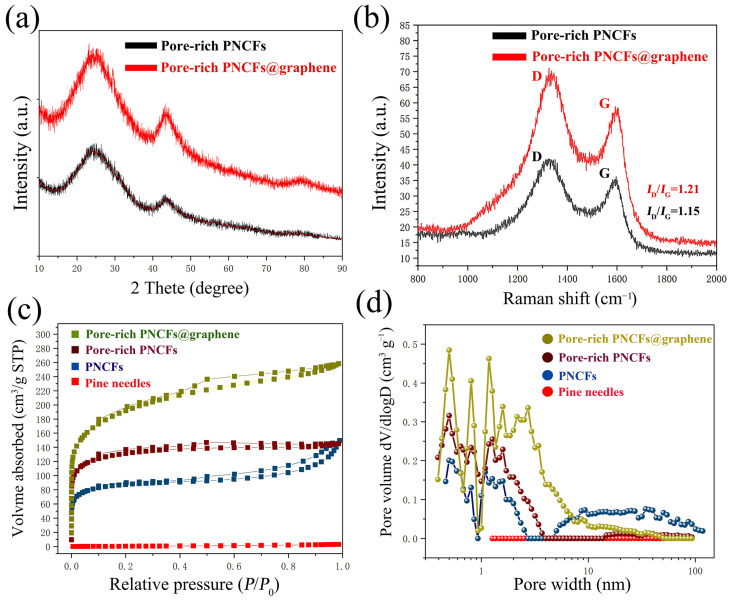
Analysis of the crystal structure, chemical composition, and pore structure of the pore-rich PNCFs@graphene composite: (**a**) XRD patterns; (**b**) Raman spectra; (**c**) N_2_ adsorption–desorption isotherms; (**d**) DFT model incremental pore volume vs. pore width.

To further study the microstructure of composite, the crystal structure of the pore-rich PNCFs and pore-rich PNCFs@graphene are monitored by XRD (Figure 4a). The XRD patterns for the pore-rich PNCFs and pore-rich PNCFs@graphene consist of two broad peaks positioned at 2*θ* = ~23.2° and 43.4°, which correspond to the (002) and (100) planes of carbon structure, respectively. By comparing the XRD patterns of pore-rich PNCFs and pore-rich PNCFs@graphene, both XRD patterns show a distinct amorphous carbon structure, and the relatively intact crystal structure of graphene after growing graphene by PECVD causes the carbon (002) and (100) peaks in pore-rich PNCFs@graphene to become narrower and sharper [35,39].

Raman spectra are employed to confirm the graphitized structure and the defect density of carbon materials. The Raman spectrum of composite shows the typical signal characteristics of graphene. The peaks located at around 1350 cm^−1^ and 1600 cm^−1^ are assigned to the characteristic D-band (defects and disorder) and G-band (graphitic) bands (Figure 4b). The G-band originates from an ideal graphitic lattice vibration mode with E_2g_ symmetry. The D-band is a typical feature of disordered graphite or crystal defects, corresponding to a graphitic lattice vibration mode with A_1g_ symmetry [40]. In addition, the intensity ratio (*I*_D_/*I*_G_) between the D and G bands is often used as a measure of the defect density in carbon materials. The larger the ratio of *I*_D_/*I*_G_, the higher the degree of defects in the carbon materials. After growing the graphene film, *I*_D_/*I*_G_ increased significantly, and the *I*_D_/*I*_G_ value increased from 1.15 (pore-rich PNCFs) to 1.21 (pore-rich PNCFs@graphene), which is caused by the graphitization edge faces (dangling bonds) exposed on the surface during the PECVD process and the additional defects generated by the active plasma [41]. The high *I*_D_/*I*_G_ value of the pore-rich PNCFs@graphene composite reveals a large disorder/defect density. The residual defects/groups of graphene may be beneficial for the electromagnetic wave absorption of the pore-rich PNCFs@graphene core-shell composite.

The large surface area of the conductor is conducive to enhancing the interaction effect between it and electromagnetic waves, so the surface area of the shielding material has been proven to be a key parameter that affects the performance of EMI shielding [42]. The surface area and porosity of pine needles, PNCFs, pore-rich PNCFs, and pore-rich PNCFs@graphene composite have been determined using the BET method. Through the pyrolysis process, the BET surface area increases from 3.0 m^2^ g^−1^ (pine needles) to 333.2 m^2^ g^−1^ (PNCFs). A combined method of KOH immersion and calcination was used to make pores for PNCFs, and the introduction of graphene by PECVD is to make the surface of PNCFs rougher, thus further increasing the specific surface area of the material. The specific surface area of the material increases from 333.2 m^2^ g^−1^ (PNCFs) to 498.1 m^2^ g^−1^ (pore-rich PNCFs) and eventually to 699.1 m^2^ g^−1^ (pore-rich PNCFs@graphene) (Table 1). Larger surface area and interface space provide more sites for electromagnetic wave scattering and multiple reflections. Nanomaterials with a larger specific area are expected to possess more superior ability to attenuate electromagnetic waves because there are more nanoparticles to be polarized to cause magnetic domain resonance and eddy loss as well as electric-thermal conversion loss when electromagnetic waves penetrate the materials. It is expected that such a high surface area of pore-rich PNCFs@graphene is effective for the attenuation of electromagnetic waves [43,44].

From the N_2_ adsorption–desorption isotherms (Figure 4c), the original pine needles almost have no adsorption capacity. By comparison, other materials, including PNCFs, pore-rich PNCFs, and pore-rich PNCFs@graphene, exhibit remarkable uptakes in the *P/P*_0_ range of 0–0.2, revealing the presence of micropores [45]. The appearance of hysteresis loops suggests the existence of mesopores [46]. The DFT model illustrates the incremental contribution of different pore sizes to pore volume, so we can infer the different pore width distributions of the material (Figure 4d). We can find that pore-rich PNCFs@graphene displays clearly stronger peak intensities compared to others. Meanwhile, the graphene grown in PECVD itself has defects ranging from sub-nanometers to several nanometers, and during the growth process, the interconnection and accumulation between graphene sheets and lamellar form pores composed of single or several layers of graphene sheets, all of which lead to wider pore distribution in the richly porous PNCFs@graphene. In addition, by comparing the brown line (pore-rich PNCFs) and the blue line (PNCFs), the peak intensity in the region of micropores increases while that in the region of mesopores and macropores decreases, suggesting that some mesopores and macropores are possibly transformed into micropores after the KOH activation treatment.

The calculated average pore size decreases from 6.55 nm (the calculated average pore size) to 2.20 nm (pore-rich PNCFs) when the calculated total pore volume increases from 0.17 cm^3^ g^−1^ (PNCFs) to 0.22 cm^3^ g^−1^ (pore-rich PNCFs) after pore formation (Table 1). The phenomena are associated with the formation of a large number of micropores by the pore formation treatment. Moreover, the graphene growth increases the total pore volume from 0.22 cm^3^ g^−1^ (pore-rich PNCFs) by 1.8 times to 0.40 cm^3^ g^−1^ (pore-rich PNCFs@graphene). This is related to the growth of graphene films and the formation of core-shell structures. Thus, the final pore-rich PNCFs@graphene has a suitable porous structure. The enhancement of the surface area and pore volume of pore-rich PNCFs@graphene and its close impedance matching with free space may accelerate the incident electromagnetic waves entering the composite material. The existence of pores in the pore-rich PNCFs@graphene can also provide channels for the penetration and transmission of electromagnetic waves to promote multiple scattering, thus further increasing the attenuation of electromagnetic waves in the composite [47]. The porous structure of pore-rich PNCFs@graphene also shows that it can be used as a lightweight EMI shielding material.

### 3.3. Electrical Conductivity and EMI Shielding Property

Theoretically, for plane waves propagating to the material surface, they will be reflected and absorbed. The remaining electromagnetic wave will penetrate the material and cause multiple reflections inside the material, thereby inducing further dissipation of electromagnetic wave energy. The EMI shielding property is measured by the EMI *SE*, which is expressed using decibels (dB) as a unit. As electromagnetic waves propagate to the surface of the material, part of the incident power (*P*_I_) will be reflected, part of the energy will be absorbed and dissipated, and the rest (*P*_T_) will pass through the shielding material [48]. The *SE*_T_ is equal to the sum of electromagnetic wave reflection *SE*_R_, electromagnetic wave absorption *SE*_A,_ and electromagnetic wave internal multiple reflection loss (*SE*_M_), expressed as:

(11) $$SE_{T}=10log\frac{P_{I}}{P_{T}}=SE_{R}+SE_{A}+SE_{M}$$

(12) $$SE_{A}=20loge^{d/\delta }\approx 8.7d\sqrt{f\pi \mu \sigma }$$

(13) $$SE_{R}\approx 39.5+10log\frac{\sigma }{2f\pi \mu }$$

(14) $$SE_{M}=20log\left(1-e^{-\frac{2d}{\delta }}\right)$$

where *σ* means the conductivity of the shielding material, *μ* is the relative permeability of the shielding material, *f* is the frequency of a certain frequency point, *d* represents the thickness of the shielding material, and *σ* represents the skin depth of the material. Reflection loss and absorption loss are the two main mechanisms of electromagnetic shielding. The internal multiple reflection loss is the electromagnetic radiation trapped between the two boundaries of the material; that is, the electromagnetic wave reflected from the second boundary returns to the first boundary, and continues to reflect from the first boundary to the second boundary, and so on. The larger surface area of the porous structure and the larger surface-interface space contribute to providing more sites for electromagnetic wave scattering and multiple reflections. To acquire more reflected waves, shielding materials must possess abundant load carriers (electrons and holes) to interact with each other.

**Figure 5:**
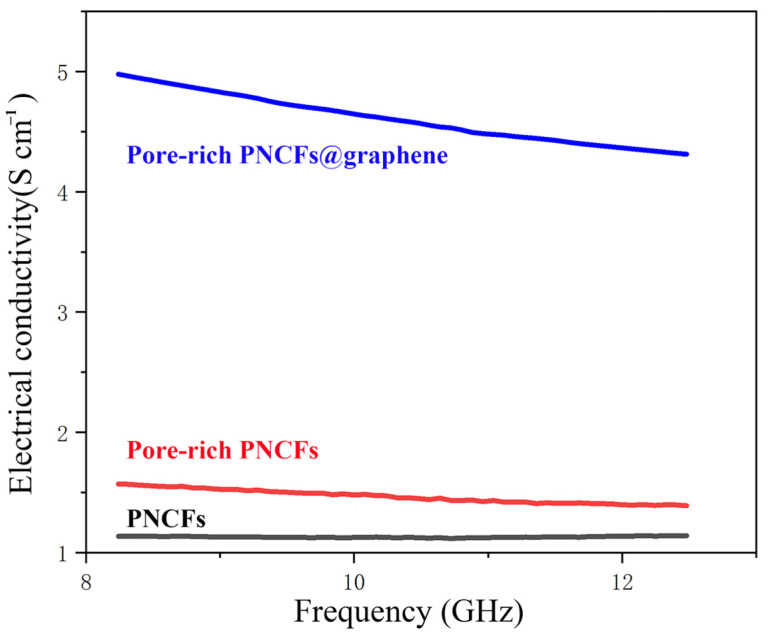
Electrical conductivity of the PNCFs, pore-rich PNCFs, and pore-rich PNCFs@graphene.

According to electromagnetic shielding theory, *SE*_A_ and *SE*_R_ are the main ways of electromagnetic shielding. Both *SE*_A_ and *SE*_R_ are proportional to the conductivity of the material. So the electrical conductivity is tightly responsible for the EMI shielding property. As the conductivity increases, the ability of the material to lose the incident radiation increases. According to the free electric theory, the electrical conductivity *σ*_r_ in an alternating electric field could be evaluated by the following Equation (15):

(15) $$\sigma_{r}\approx 2\pi f\varepsilon_{0}\varepsilon \prime \prime$$

where *f* is frequency, *ε*_0_ is the permittivity of free space (*ε*_0_ = 8.854 × 10^−12^ F/m), and *ε*″ is the imaginary permittivity [49,50]. The electrical conductivity of the PNCFs, pore-rich PNCFs, and pore-rich PNCFs@graphene are depicted in Figure 5. The electrical conductivity of the PNCFs that are pyrolyzed from the pine needle product reaches 1.13 S cm^−1^, and the conductivity of the pore-rich PNCFs rises to 1.57 S cm^−1^. The difference between them is mainly attributed to the construction of conductive networks. The conductive networks promote the movement of charge carriers and provide more pathways for the transport of electrons in the pore-rich PNCFs, thereby improving the electrical conductivity of pore-rich PNCFs. The unique graphene “shell” in the composite is very important in improving conductivity. Due to the high conductivity of the graphene shell in the pore-rich PNCFs@graphene, the pore-rich PNCFs@graphene reaches 4.97 S cm^−1^ at 8.2 GHz, 216.6% higher than that of the pore-rich PNCFs (1.57 S cm^−1^). High electrical conductivity facilitates the efficient mobilization of charge carriers in the pore-rich PNCFs@graphene, which leads to the promotion dissipation of incident electromagnetic waves on the surface of the core-shell composite and improves the EMI *SE* of the composite.

**Figure 6:**
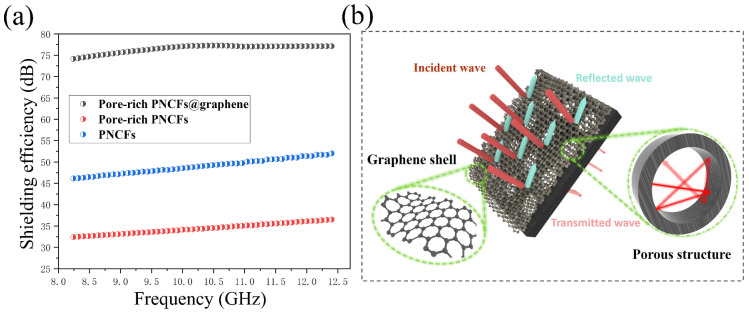
(**a**) *SE*_T_ values of the PNCFs, pore-rich PNCFs, and pore-rich PNCFs@graphene; (**b**) EMI shielding mechanism of the pore-rich PNCFs@graphene core-shell composite.

To investigate the EMI shielding property of the pore-rich PNCFs@graphene, the composite is tested in the frequency range of 8.2–12.4 GHz (X-band, a quite common band for military and communication, such as radar, telephones, and TVs). Generally, the total EMI shielding effectiveness (*SE*_T_) is the summation of all attenuating functions. The pine needles present an extremely low *SE*_T_ value, indicating that pine needles almost have no EMI shielding ability. By contrast, the PNCFs deliver a much higher *SE*_T_ maximum value of ~36 dB. The maximum value of *SE*_T_ rises to ~52 dB for the pore-rich PNCFs. As the graphene is introduced, the maximum value of *SE*_T_ increases to ~77 dB for the pore-rich PNCFs@graphene (Figure 6a). Such a high EMI *SE* proves the excellent effect of this unique core-shell and porous structure pore-rich PNCFs@graphene composite on electromagnetic shielding. The possible shielding mechanism is shown in Figure 6b. In addition, this high *SE* is superior to that of many recently reported biomass-based EMI shielding products, such as the Cs/epoxy (28 dB) [14], PEI/PA@AgNWs (32.9 dB) [16], honeycomb-like lignin-based carbon/graphene foams (28.5–70.5 dB) [51], loofah sponge-derived carbon/paraffin/urethane (32 dB) [52], epoxy/CNT sponge (33 dB) [53], Fe_3_O_4_@reduced graphene oxide (rGO)/natural rubber (37 dB) [54], rGO/polystyrene (45.1 dB) [55], multi-walled carbon nanotubes (MWCNT)/water-borne polyurethane (49.2 dB) [56], ALC based on sugarcane (51 dB) [15], SC-Co-G (55 dB) [21], and cotton fiber-derived carbon/CoFe alloy (~62 dB) [57], as summarized in Table 2. To study the effect of structural damages of the carbonized needles on the EMI shielding property, we blended the powder-like pore-rich PNCFs@graphene sample with paraffin, and the sample shows a similar EMI SE value, as compared to that of the sample based on non-grinding pore-rich PNCFs@graphene. The maximum difference between them is only 2 dB (Figure 6a). Therefore, the common physical treatment (such as grinding) on the carbonized needles will not notably affect the EMI shielding property.

EMI shielding efficiency is usually expressed as a percentage (%) to indicate the ability of a material to block waves. The percentage shielding effect *X*% is the percentage of the difference between the energy in the shielding field and the energy outside the field relative to the energy in the field. For example, 99%, that is, the radiation intensity of the radiation source is shielded by 99%. EMI shielding effectiveness (dB) is converted into EMI shielding effectiveness (%) using Equation (16) as follows:

(16) $$Shielding\;efficiency(\%)=100-\left(\frac{1}{100^{\frac{SE}{10}}}\right)\times 100\%$$

After substituting the data into the calculation, the pore-rich PNCFs@graphene shows a shielding efficiency of 99.9999%. This shows that the composite can shield most electromagnetic waves.

To estimate the mechanism of material dissipating electromagnetic waves more accurately, the EMI absorptivity *A* (*A* = *SE*_A_/*SE*_T_) of PNCFs, pore-rich PNCFs, and pore-rich PNCFs@graphene are further analyzed. *A* can be approximately expressed as [58,59]:

(17) $$A=\frac{SE_{A}}{SE_{total}}\approx \frac{SE_{A}}{SE_{A}+SE_{R}}∼\frac{d\sqrt{u_{r}\omega \sigma_{AC}}}{\log\frac{\sigma_{AC}}{u_{r}\omega }}$$

**Figure 7:**
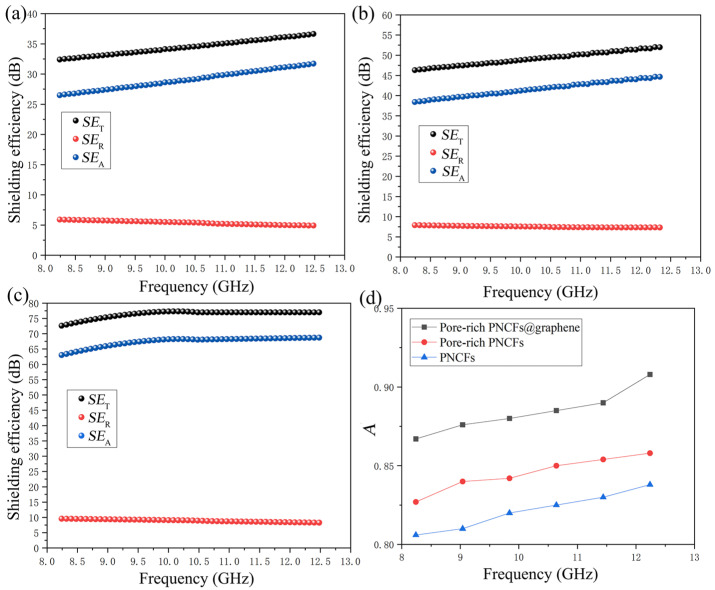
*SE*_T_, *SE*_A,_ and *SE*_T_ values of the (**a**) PNCFs, (**b**) pore-rich PNCFs, and (**c**) pore-rich PNCFs@graphene, respectively; (**d**) the *A* value of X-band PNCFs, pore-rich PNCFs and pore-rich PNCFs@graphene.

*SE*_A_ and *SE*_R_ can be expressed by Equations (18) and (19):

(18) $$SE_{A}=20d\sqrt{\frac{u_{r}\omega \sigma_{AC}}{2}}\cdot \log e$$

(19) $$SE_{R}=10\log\left(\frac{\sigma_{AC}}{16\omega u_{r}\varepsilon_{0}}\right)$$

where *d* is the thickness of the materials, *μ*_r_ is the magnetic permeability, *ω* is the angular frequency, *σ*_AC_ is the frequency-dependent conductivity. The *SE*_T_, *SE*_R,_ and *SE*_A_ values of the PNCFs, pore-rich PNCFs, and pore-rich PNCFs@graphene are presented in Figure 7a–c. With the formation of porous and core-shell structure, the maximum *A* increases from 83.6% for PNCFs to 85.8% for the pore-rich PNCFs and eventually to 90.8% for the pore-rich PNCFs@graphene (Figure 7d), shows absorption-dominating shielding mechanism. The maximum *A* of the pore-rich PNCFs@graphene in this work is 90.8%, which is much higher than the maximum value of other shielding materials in the X-band [60,61]. Clearly, combining Equations (7)–(9), the growth of *A* is due to *σ*_AC_, and *σ*_AC_ is clearly attributed to graphene. This core-shell structure with graphene as the shell introduces an interface with impedance mismatch, and the multilayer nanostructure provides a new reflection interface for electromagnetic waves so that electromagnetic waves are first dissipated on the surface of the graphene shell. The incoming electromagnetic wave is trapped in the porous structure and attenuated by the absorption and multiple reflections between the core and shell in pore-rich PNCFs@graphene. This highly porous structure can significantly enhance internal multi-reflection, and the internal absorption in the pores can be repeatedly used to enhance *A*. Moreover, due to the larger surface area and surface-interface space of the pore-rich PNCFs@graphene, there are more sites for the scattering and multiple reflections of electromagnetic waves. The remaining electromagnetic waves will produce multiple internal reflections in the porous structure of the pore-rich PNCFs@graphene, which will cause the electromagnetic energy to be further dissipated and scattered until most of the electromagnetic waves are absorbed. In addition, the porous core-shell structure provides abundant polarization centers, thereby capturing electromagnetic waves and promoting attenuation through additional dielectric loss. Therefore, the higher absorption loss is mainly attributed to the conductive network structure formed by graphene and carbon fibers, a time-varying electromagnetic field-induced current occurs therein, and induced current in the resistance network and converted into heat energy and multiple reflections of electromagnetic waves in the porous structure, resulting in the rapid decay of electromagnetic waves. In our work, the maximum *A* of pore-rich PNCFs@graphene reaches 90.8%, which is much higher than that of congeneric porous shielding materials. The absorption-based shielding mechanism contributes to stealth applications (such as warcrafts, missiles, and stealth wears).

**Figure 8:**
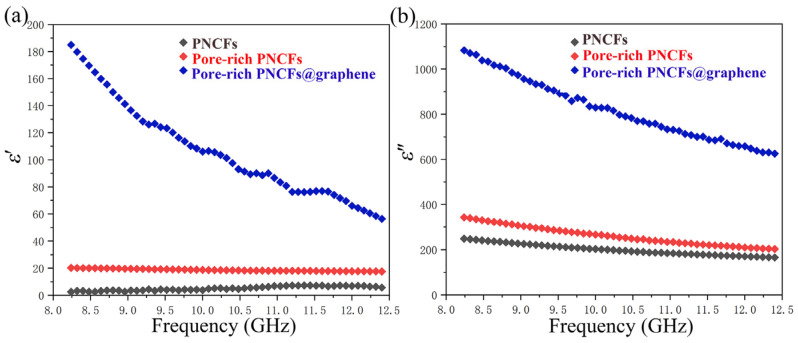
Complex permittivity of the PNCFs, pore-rich PNCFs, and pore-rich PNCFs@graphene. (**a**) Real part; (**b**) imaginary part.

The electromagnetic property of the pore-rich PNCFs@graphene is determined by complex permittivity (*ε* = *ε*′ − i*ε*″) and permeability (*μ* = *μ*′ − i*μ*″). The real part of the electromagnetic parameters (*ε*′, *μ*′) is a measure of the polarization amount and means the capability for electric or magnetic energy storage, while the imaginary part (*ε*″, *μ*″) represents the energy loss ability. As seen in Figure 8a, the *ε*′ decreases with the increase in frequency. This behavior is raised by the lag of the induced electric field reversed external electric field at high frequency [62]. After the graphene film is grown on the pore-rich PNCFs@graphene surface, the value of *ε*′ increases obviously. The result indicates the remarkably improved electric energy storage capacity of the composite.

Figure 8b shows the imaginary part of the complex permittivity of the PNCFs, pore-rich PNCFs, and pore-rich PNCFs@graphene. Similarly, the *ε*″ value improves significantly after the graphene film is grown. The higher permittivity leads to a higher dielectric loss due to the presence of graphene film on the surface of pore-rich PNCFs. In addition, because of the weak magnetic property of carbon materials, the magnetic permeabilities of *μ*′ and *μ*″ are very low, almost negligible.

### 3.4. Mechanical Properties

**Figure 9:**
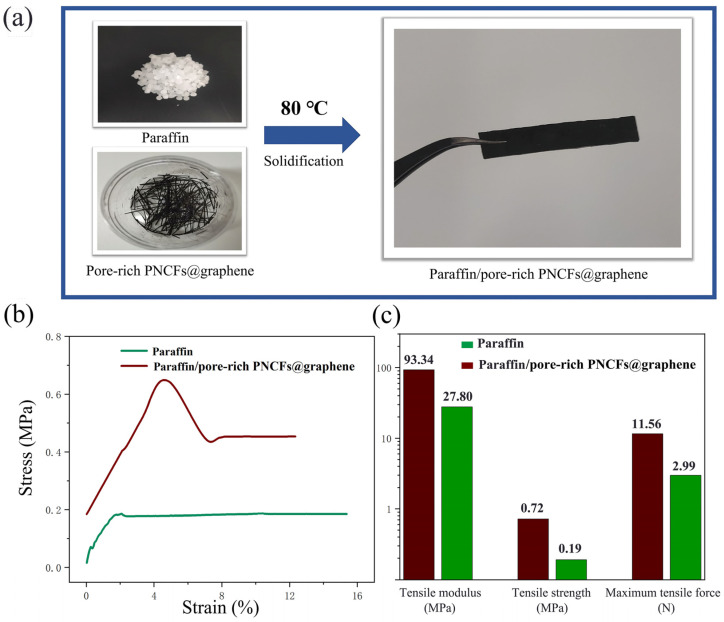
(**a**) Preparation of the paraffin/pore-rich PNCFs@graphene samples for tensile experiments. (**b**) Stress-strain relationships of the pure paraffin and paraffin/pore-rich PNCFs@graphene. (**c**) Tensile modulus, tensile strength, and maximum tensile force of the pure paraffin and paraffin/pore-rich PNCFs@graphene.

Figure 9a shows the paraffin/pore-rich PNCFs@graphene specimen (40 mm × 8 mm × 2 mm) for tensile experiments. We compare the tensile behavior of the pure paraffin and paraffin/pore-rich PNCFs@graphene samples to study the effect of the pore-rich PNCFs@graphene on the mechanical performance of the paraffin matrix. The tensile stress-strain curves of the pure paraffin and paraffin/pore-rich PNCFs@graphene are shown in Figure 9b. The curves and data reveal that the pure paraffin matrix presents distinct plastic characteristics. By calculation, the paraffin/pore-rich PNCFs@graphene shows the tensile modulus, tensile strength, and maximum tensile force of 93.34 MPa, 0.72 MPa, and 13.56 N, respectively. These data are 3.36, 3.79, and 4.54 times those of the paraffin (Figure 9c). The main reason for these increases is the decrease in the amount of low-molecular-weight paraffin due to the loading of the pore-rich PNCFs@graphene fillers. Paraffin itself has very low tensile strength, and paraffin crystals in the amorphous phase easily act as defect spots for the initiation and propagation of stress cracking. In addition, the high plasticity of paraffin can fill the voids, contributing to the interaction between the pore-rich PNCFs@graphene fillers and the paraffin matrix. As a result, the bond strength at the interface between them is improved.

## 4. Conclusions

From the results obtained in this investigation, the following conclusions can be drawn:

- By activation and PECVD treatment, the specific surface area of the material increases from 333.2 m^2^ g^−1^ (PNCFs) to 498.1 m^2^ g^−1^ (pore-rich PNCFs) and eventually to 699.1 m^2^ g^−1^ (pore-rich PNCFs@graphene). Larger surface area and interface space provide more sites for electromagnetic wave scattering and multiple reflections.
- After the growth of graphene with the help of PECVD, total pore volume increased 1.8-fold from 0.22 cm^3^ g^−1^ (pore-rich PNCFs) to 0.40 cm^3^ g^−1^ (pore-rich PNCFs@graphene). This porous structure will facilitate the multiple reflections of the incident electromagnetic waves.
- The generation of conductive networks promoted by the growth of graphene, the as-prepared core-shell structure has a greatly improved conductivity from 1.13 S cm^−1^ (PNCFs) to 4.97 S cm^−1^ (pore-rich PNCFs@graphene).
- Because of the combination of high electrical conductivity and unique core-shell porous structure, the PNCFs@graphene composite delivers a high EMI shielding effectiveness of ~77 dB, much higher than its precursors (PNCFs: ~36 dB; pore-rich PNCFs: ~52 dB), respectively.
- Due to the enhanced surface area and pore volume of porous PNCFs@graphene to promote the dissipation of electromagnetic waves in the composite, the absorption ratio increases from 83.6% for PNCFs to 85.8% for the pore-rich PNCFs and eventually to 90.8% for the pore-rich PNCFs@graphene. This absorption-based shielding mechanism will have great potential for developing stealth materials.

## Figures and Tables

**Table 1:** BET surface area, calculated pore volume and average pore size of pine needles, PNCFs, pore-rich PNCFs, and pore-rich PNCFs@graphene composite.

Sample	BET Surface Area (m^2^/g)	Total Pore Volume (cm^3^/g)	Average Pore Size (nm)
Pine needles	3.0	0.0047	6.22
PNCFs	333.2	0.17	6.55
Pore-rich PNCFs	498.1	0.22	2.20
Pore-rich PNCFs@graphene	699.1	0.40	2.83

**Table 2:** Recently reported *SE*_T_ value of some biomass-based EMI shielding materials.

Samples	*SE*_T_ (dB)	Electrical Conductivity (S cm^−1^)	Frequency (GHz)	Ref.
Cs/epoxy	28	~0.01	8.2–12.4	[14]
Lignin-based carbon/graphene foams	28.5–70.5	~0.98	8.2–12.4	[51]
Loofah sponge-derived carbon/paraffin/urethane	32	-	8.2–12.4	[52]
PEI/PA@AgNWs	32.9	-	8–18	[16]
Epoxy/CNT sponge	33	5.16	8–12	[53]
Fe_3_O_4_@rGO/natural rubber	37	~0.01	8.2–12.4	[54]
rGO/polystyrene	45.1	0.43	8.2–12.4	[55]
MWCNT/water-borne polyurethane	49.2	0.44	8.2–12.4	[56]
ALC based on sugarcane	51	0.09	8.2–12.4	[15]
SC-Co-G	55	~0.22	12.4–18	[24]
Cotton fiber-derived carbon/CoFe alloy	~62	-	8.2–12.4	[57]
PNCFs	36	1.13	8.2–12.4	This work
Pore-rich PNCFs	52	1.57	8.2–12.4	This work
Pore-rich PNCFs@graphene	77	4.97	8.2–12.4	This work

## Data Availability

Not Applicable.
